# Fabrication of Artificial Nerve Conduits Used in a Long Nerve Gap: Current Reviews and Future Studies

**DOI:** 10.3390/bioengineering11040409

**Published:** 2024-04-22

**Authors:** Ryosuke Kakinoki, Yukiko Hara, Koichi Yoshimoto, Yukitoshi Kaizawa, Kazuhiko Hashimoto, Hiroki Tanaka, Takaya Kobayashi, Kazuhiro Ohtani, Takashi Noguchi, Ryosuke Ikeguchi, Masao Akagi, Koji Goto

**Affiliations:** 1 Department of Orthopedic Surgery, Kindai University Hospital, 377-2 Oono-higashi, Osaka-sayama 589-8511, Japan; 2 Department of Orthopedic Surgery, Kansai Electric Power Hospital, 2-1-7 Fukushima, Fukushima-ku, Osaka City 553-0003, Japan; 3 Department of Orthopedic Surgery, Graduate School of Medicine, Kyoto University, 54 Shougoin-Kawahara-cho, Sakyo-ku, Kyoto 606-8507, Japan

**Keywords:** artificial nerve, vascularity, fibrin matrix, scaffolds, stem cells

## Abstract

There are many commercially available artificial nerve conduits, used mostly to repair short gaps in sensory nerves. The stages of nerve regeneration in a nerve conduit are fibrin matrix formation between the nerve stumps joined to the conduit, capillary extension and Schwann cell migration from both nerve stumps, and, finally, axon extension from the proximal nerve stump. Artificial nerves connecting transected nerve stumps with a long interstump gap should be biodegradable, soft and pliable; have the ability to maintain an intrachamber fibrin matrix structure that allows capillary invasion of the tubular lumen, inhibition of scar tissue invasion and leakage of intratubular neurochemical factors from the chamber; and be able to accommodate cells that produce neurochemical factors that promote nerve regeneration. Here, we describe current progress in the development of artificial nerve conduits and the future studies needed to create nerve conduits, the nerve regeneration of which is compatible with that of an autologous nerve graft transplanted over a long nerve gap.

## 1. Introduction

Bridging transected nerve stumps using a tube-like material is called tubulation. Currently, the distance across which axons can regenerate through silicone tubulation in rat sciatic nerves is limited to about 10 mm [1]. In addition, the numbers of regenerated axons are fewer, and their diameters are smaller than those seen with an autologous nerve graft [2,3].

The process of nerve regeneration in tubulation is well described by Williams et al. [4]. The first stage of nerve regeneration in a nerve conduit is the formation of a fibrin matrix within the chamber space between the transected nerve stumps joined to either end of the conduit. The next stage is capillary extension associated with Schwann cell migration into the fibrin matrix from both transected nerve stumps. Following capillary extension and Schwann cell migration, axons extend from the proximal nerve stump into the fibrin matrix.

To create a nerve conduit that can be a successful alternative to an autologous nerve graft, the nerve regeneration distance needs to be longer, and the numbers and diameters of axons must be increased. To achieve these goals, facilitation of the abovementioned stages of intrachamber nerve regeneration is essential.

We have attempted to obtain successful nerve regeneration for a 20 mm interstump gap in rat sciatic nerves using artificial nerves by promoting intrachamber fibrin matrix formation, capillary extension, Schwann cell migration and axon extension. We used vascularity, cells, growth factors and a scaffold, which are the four essential factors in tissue engineering. Nerve regeneration across a 20 mm gap in rats has been reported to be equivalent to a 60 mm gap in primates [5]. In this article, we describe important neurological and bioengineering concepts and related techniques needed to create a successful artificial nerve conduit that could be an alternative to an autologous nerve graft over a long interstump gap.

## 2. Promotion of Fibrin Matrix Formation

The first stage of nerve regeneration is fibrin matrix formation in the conduit space between the nerve stumps joined to the ends of the conduit [4]. The distance across which axons can regenerate is determined by the length of the fibrin matrix through which capillaries can extend. Primarily, there are two options to create the environment in which the fibrin matrix can be formed over a long distance within the conduit. A substrate (scaffold) that maintains the structure of the fibrin matrix over the distance could be transplanted into the chamber space [6,7,8] or the nature of the conduit chamber could be modified to be more adherent to the fibrin matrix [9,10].

### 2.1. Transplantation of an Intratubular Scaffold

**Figure 1:**
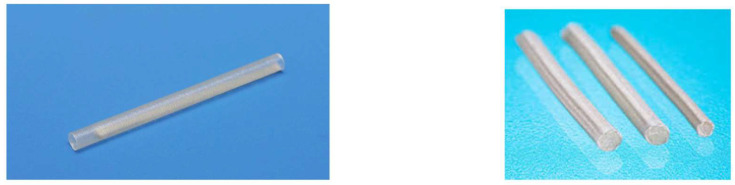
Commercially available artificial nerves. **Left**: Renerve^®^ (Nipro, Osaka, Japan); **right**: Nerbridge^®^ (Toyobo, Osaka, Japan). The conduit chambers are occupied by collagen materials in both nerve conduits.

A fibrin matrix is not formed when the interstump gap is too long or in a conduit through which the fibrin matrix can leak out through its wall. Additional substrate is needed in the chamber space to obtain successful fibrin matrix formation over a long distance [6,7,8]. In some commercially available nerve conduits with capillary permeability, such as Nerbridge^®^ (Toyobo Co., Ltd., Osaka, Japan: https://www.toyobo-global.com/system/files/News_Release/201902/press20180820A.pdf accessed on 1 March 2019) and Renerve^®^ (Nipro Co., Ltd., Osaka, Japan: https://renerve.com.au/ accessed on 1 May 2022), collagen sponges or collagen fibers are transplanted into the conduit chamber as scaffolds for fibrin matrix formation and axon extension through the conduit (Figure 1).

### 2.2. Use of a Hydrophilic Tube

A conduit, the inner surface of which is hydrophilic, is advantageous for maintaining the structure of the fibrin matrix within that nerve conduit. A negatively charged silicone plate, onto which negative ions have been injected, shows stronger hydrophilicity than nontreated silicone plates [9]. By using negatively charged silicone conduits prepared with a negatively charged carbon ion injection on the inner surface of the conduits, nerve regeneration was extended to 15 mm in a rat sciatic nerve model [10]. Recently, a nerve conduit made of aggregated fibroblast spheres using a three-dimensional computer printing technique demonstrated improved nerve regeneration [11,12]. Fibrin matrix adherence might have been facilitated by the hydrophilic nature of the conduit wall made by the cell spheres.

## 3. Facilitation of Intratubular Vascularity

Axon growth cones and capillary tip cells share common signal cues in nerve regeneration [13]. In tissue regeneration, neuropilin and ephrin receptors are expressed in both vascular and nerve tissue [14]. Axon extension is thus accompanied by capillary extension, and it is highly likely that improved vascularization within the chamber space is followed by a promotion of axon extension. In a tubulation model using a silicone tube, capillaries extended into the fibrin matrix only from the nerve stumps joined to either end of the tube [4]. Previous researchers have attempted to promote capillary extension in tubulation in several ways: by transplantation of a thin vascular pedicle into the conduit lumen [6,7,15,16,17,18,19]; by the use of capillary permeable tubes [8,20,21]; by the creation of prefabricated vascularized tubes [22,23]; and by intrachamber administration of chemical factors promoting capillary proliferation [24,25].

### 3.1. Transplantation of an Intratubular Vascular Pedicle

**Figure 2:**
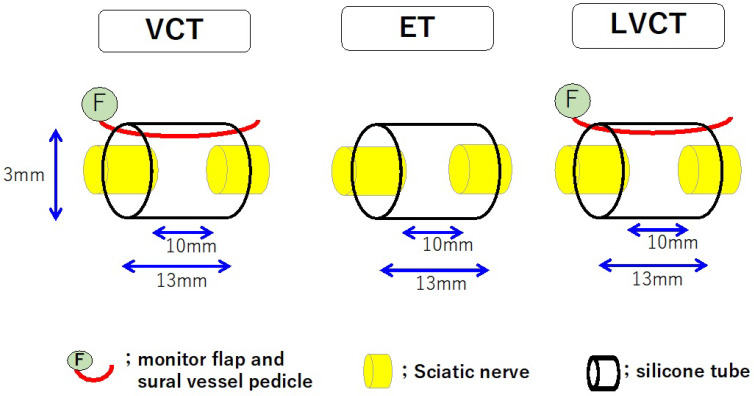
Schematic diagrams of three kinds of nerve conduit tubes: VCT (vessel-containing tube); ET (empty tube); and LVCT (ligated vessel-containing tube). When a vessel-containing tube (VCT) was created, a myocutaneous flap supplied by the sural vessels was elevated from the lower hind limb and turned proximally. The sural vessel pedicle was inserted into the chamber space through a longitudinal slit of the silicone tube, which was sealed with silicone liquid after the pedicle insertion. Each tube was transplanted between the transected sciatic nerve stumps, leaving a 10 mm gap. In LVCTs, the sural vessels inserted into the conduits were ligated.

A silicone tube containing a blood vessel pedicle was created to promote vascularity in the chamber space [15]. Using rats, a sural vessel pedicle was elevated from the lower hind limb and turned proximally. The vascular pedicle was inserted into the silicone tube transplanted between the transected sciatic nerve stumps through a longitudinal slit in the silicone tube. The slit was sealed with silicone liquid after pedicle insertion to create a vessel-containing tube (VCT). As controls, two other conduit models were created. One was an empty tube (ET), a silicone tube, to the ends of which the transected nerve stumps were joined. The other was a ligated vessel-containing tube (LVCT). The LVCT contained a sural vessel pedicle in the chamber; however, the pedicle was ligated and had no blood flow (Figure 2). Using a 10 mm interstump gap model in rat sciatic nerves, nerve regeneration was significantly promoted in VCTs at 6 and 12 weeks compared to the nerve regeneration in ETs and LVCTs. However, no significant difference was found among the three tubes at 24 weeks. A microangiographic study demonstrated that a vascular network was rapidly formed in the fibrin matrix around the sural vessel pedicle in VCTs at three weeks [15]. The rapid nerve regeneration in VCTs may result from the prompt capillary formation in the chamber space of VCTs. Using VCTs, the distance across which axons regenerated was extended to 25 mm in rat sciatic nerves [17]. Additional vascularity within the chamber space accelerated the rate of nerve regeneration and extended the axon regeneration distance. However, it did not increase the number or diameter of regenerated axons [15]. This indicates that factors other than intrachamber vascularity are necessary to increase axon number and diameter.

### 3.2. Capillary Permeable Nerve Conduits

Inserting a blood vessel pedicle into the chamber space is technically demanding. Nerve conduits that have porous structures in their walls through which capillaries can pass introduce vascularity into the chamber space more easily than blood vessel transplantation. However, these conduits are associated with a risk of allowing scar tissue invasion into the chamber space and permitting leakage of the fibrin matrix and neurochemical factors from the chamber, leading to failure of nerve regeneration within the conduits. When capillary permeable conduits are used, a substrate is required to keep the fibrin matrix inside the conduit. In some commercially available tubes with capillary permeability, such as Nerbridge^®^ and Renerve^®^, collagen sponge or collagen fibers are transplanted in the chamber space and might be used as a scaffold for fibrin matrix formation. However, collagen substrates in the chamber carry the risk of physically interfering with axon extension (Figure 1).

**Figure 3:**
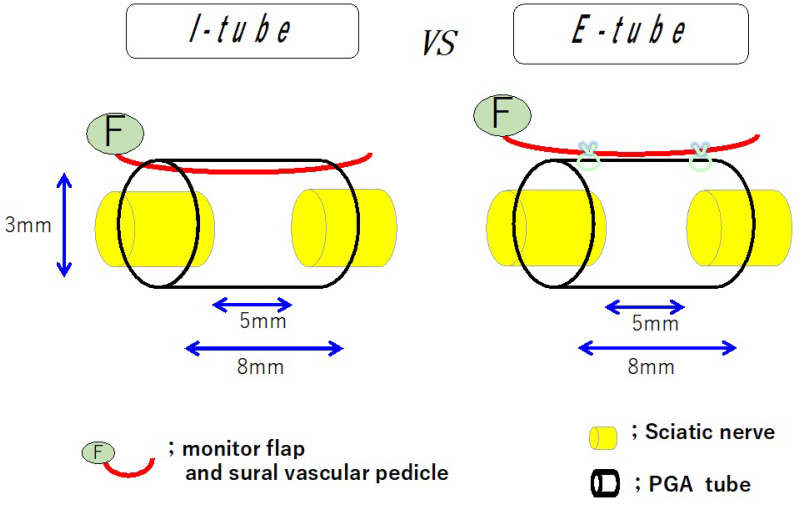
Schematic diagrams of the I-group (a sural vessel pedicle was passed through the chamber space of a PGA conduit) and the E-group (a sural vessel pedicle was attached to the outer surface of a PGA conduit). The interstump gap was set at 5 mm.

Nerbridge^®^ comprises an outer cylinder made of a woven polyglycolic acid (PGA) fiber mesh and an inner core made of collagen sponge. The outer cylinder of Nerbridge^®^ is permeable to particles smaller than 600 kDa [8]. To examine the cell permeability of the outer cylinder of Nerbridge^®^ (a PGA conduit), two conduit models were created in a previous study [8]. One was an 8 mm long PGA conduit, the chamber space of which contained a sural vessel pedicle (I-group). The other was an 8 mm long PGA conduit, to the outer surface of which a sural vessel pedicle was attached (E-group). In both groups, each conduit was wrapped with a 20 × 20 mm silicone sheet to shut down the vascularity without the nerve stumps joined to each tube and the transplanted sural vessel pedicle (Figure 3). A rat endothelial cell antigen 1 immunostaining study demonstrated that there was no significant difference in intrachamber capillary formation between the I- and E-groups at four weeks. Electrophysiologic and histomorphometric studies showed that no significant difference was found in nerve regeneration between both groups at 12 weeks. Histological examination demonstrated no remarkable scar tissue invasion into the tubular lumen in the E-group. The outer cylinder of Nerbridge^®^ allowed capillaries to extend into the chamber space and prevented scar tissue from entering the space [8]. The pore size of the PGA fiber mesh of Nerbridge^®^ might be crucially important to endothelial cell permeability and inhibition of fibroblast invasion [8,20,21]. Notably, the sizes of these cells may change depending on the cellular environment [26,27,28]. Thus, the detailed mechanism of cell selection through the PGA fiber mesh wall is not well understood and further study is needed. The optimal core size of the wall of a conduit with capillary permeability and scar tissue inhibition should also be investigated.

### 3.3. Prefabricated Vascularized Nerve Conduits

To create a prefabricated vascularized tube in animal models, a silicone rod is transplanted in fully vascularized tissue. Two to three months after transplantation, the rod becomes wrapped with vascularized connective tissue. The rod is then removed while maintaining vascular continuity with the tube-like tissue created around the rod. Tube-like vascularized connective tissue is thus created. Promotion of nerve regeneration through vascularized prefabricated tubes has been reported by several authors [22,23]. Note that prefabricated vascularized tubes may take a long time to be created and are prone to structural weakness.

### 3.4. Chemical Factor Application

Intrachamber transplantation of chemical factors, including growth factors such as glial cell-line-derived neurotrophic factors [29], vascular endothelial growth factor [24,25], or basic fibroblast growth factor [30,31], may be an option to promote intrachamber vascularity. However, the optimal timing or methods to deliver the factors into the chamber space have not been well defined. In addition, how to prevent leakage of the implanted chemical factors from the chamber space is also a problem yet to be solved.

## 4. Intratubular Cell Transplantation

Autologous nerve grafts contain an abundance of Schwann cells. After harvesting the nerve graft, the functions of Schwann cells in the graft change from conveying electric signals (static phase) to producing various neurochemical factors through the massive proliferation of cells (proliferative phase). Schwann cells in the proliferative phase in an injured nerve can form a Bünger band through which the regenerating axons extend and secrete several neurochemical factors, which can promote nerve regeneration [32]. Schwann cells transplanted into the conduits are expected to function like Schwann cells in autologous nerve grafts. However, it is still unknown whether cultured Schwann cells would be able to switch function from conveying electric signals to secreting neurochemical factors in the same way as Schwann cells of autologous nerve grafts. Moreover, it takes a long time to increase the number of Schwann cells in an in vitro culture [20,29].

Recently, stem cells have attracted attention as a cell source in tissue engineering. Stem cells aggregate at the site of injured tissue [33,34,35] to produce neurochemical factors [36,37,38] and transdifferentiate into several different cell types as determined by the transplantation environment [39,40,41,42]. Some researchers have noted that stem cells transplanted within a nerve conduit are transdifferentiated into cells with neural or glial characteristics in the conduits [38,42]. However, there are several views about the mechanism of transdifferentiation of stem cells at the recipient site [43], suggesting that the main role of transplanted stem cells is the production of chemical factors. Schwann cells [20,29,44], induced pluripotent stem cells [45,46], bone marrow-derived mesenchymal stem cells (BMSCs) [6,7,8,18,19,42] and adipose tissue-derived stem cells (with or without gene modification) [25,47] are often used as cell sources for intrachamber transplantation. The cell class most suitable for peripheral nerve regeneration in tubulation is still unknown.

## 5. Chemical Factor Administration

Various chemical factors have been administered at the site of nerve regeneration using different delivery systems, such as local or systemic administration of neurochemical factors [31,48,49], viral vectors encoding chemical factors capable of facilitating nerve regeneration [24,29], exosomes containing some proteins, and genetic materials that have the ability to promote nerve regeneration [50,51]. How to administer effective doses of factors into the chamber is an aspect that should be considered in the delivery of these factors [48]. Different phases of nerve regeneration need different factors. Further studies are needed to investigate how and when each neurochemical factor should be applied to the chamber space to promote nerve regeneration.

Intrachamber stem cell transplantation may be a suitable method for the delivery of neurochemical factors into the chamber space. Stem cells transplanted into the chamber space can release neurochemical factors for nerve regeneration [36,37,38]. Several current studies are attempting to induce type-2 macrophages in the chamber space to promote nerve regeneration. Type-2 macrophages are known to produce various chemical factors, some of which are essential for nerve regeneration [13,32].

## 6. Scaffold Transplantation

An intratubular scaffold should act to keep the fibrin matrix in the chamber space. Recently, attention has been focused on decellularized allogenic nerve basal lamellae (DANBLs) as a scaffold for peripheral nerve regeneration [6,7,8,52]. DANBLs exhibit a honeycomb structure [8]. Fibrin matrix is formed in each honeycomb space surrounded by the basal lamellae. It is known that DANBLs preserve extracellular matrix, including laminin, which accelerates nerve regeneration [6,7,8,52].

DANBLs can be created by a thermal method [6,7,53], irradiation [54], and chemical surfactant techniques [8,52,55,56]. DANBLs made by chemical surfactants [55,56] contain less cellular debris and degenerated nerve tissue compared with those made by the thermal method. Thermally created DANBLs contain more genomic DNA than DANBLs created using the surfactant extraction method [8]. Thus, DANBLs made by the surfactant extraction method might be associated with a lower chance of pathological organism contamination from donors than thermally created DANBLs [8].

Some stem cells can modulate the immune reaction in tissue transplantation [57,58,59]. The immunogenicity of DANBLs may be reduced by BMSC transplantation. DANBLs seeded with BMSCs transplanted in a nerve conduit demonstrate minimum immunogenicity to a degree that is almost comparable with that of a fresh autologous nerve graft and significantly lower than that of a fresh allogenic nerve graft [6,7,8]. Thus, from the perspective of intrachamber nerve regeneration, immunogenicity and lack of disease transmission from donors to recipients, DANBLs created by chemical surfactants appear to be the best candidates for intratubular scaffolds fostering nerve regeneration.

Previous studies reported that axons can be successfully regenerated through decellularized autologous muscle basal lamellae (DMBLs) [60,61]. DMBLs do not carry the risks of immunogenicity or disease transmission associated with donor–recipient transplantation. These studies demonstrate that the basal lamellae of muscle tissue remain in the DMBLs and that the direction of the muscle fibers of the transplanted muscles affect nerve regeneration through the conduits containing DMBLs. Our preliminary study (data not yet presented) using a rat sciatic nerve model with a 20 mm interstump gap showed that there were significantly more axons with greater diameters in VCTs containing DANBLs than VTCs with DMBL transplantation 12 weeks after transplantation. This indicates that basal lamellae do not always effectively promote axon regeneration. The structural morphology of the basal lamellae might be closely related to axon regeneration capability. Because DANBLs originate from nerve tissue, their structure might be more suitable for Schwann cell migration and axon extension than that of DMBLs. The details are still unknown.

When stem cells are transplanted within the chamber, the scaffold in the chamber may also function to anchor the transplanted cells within the chamber space.

## 7. Structure of Nerve Conduits

Nerve conduits should be soft and pliable [8]. Solid tubes such as silicone tubes are not suitable for clinical use because solid tubes are associated with a risk of protrusion through the overlying skin when they are transplanted in the joint portions. Conversely, if the mechanical strength of the conduits is weak, the tubular space might collapse or become kinked by compression forces produced by the surrounding tissue. Nerve conduits should be biodegradable. Biodegradable conduits are not associated with removal of the conduits after completion of nerve regeneration. Regarding biodegradation, which leads to loss of tubular structure, the time at which it starts and its duration may also influence nerve regeneration. The rate of the biodegradation should be adjusted according to the interstump gap. In some artificial conduits made of PGA or polylactic acid (PLA) containing ε-caprolactone, the rate of the biodegradation of the conduits can be controlled by the content percentages of ε-caprolactone to PGA or PLA [19].

## 8. Our Nerve Conduit

**Figure 4:**
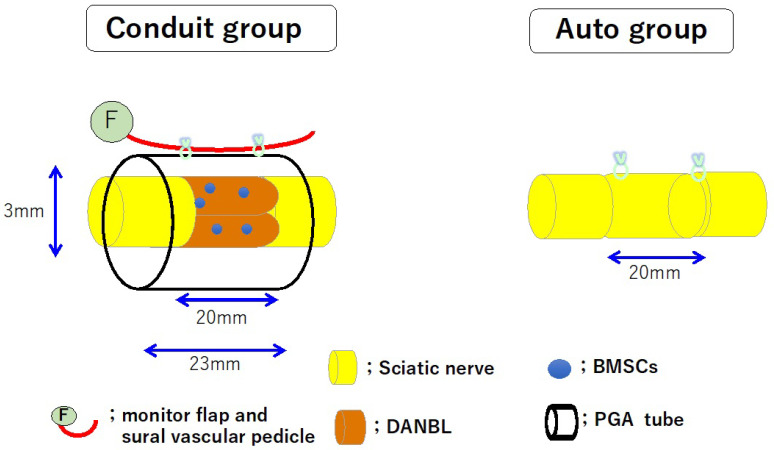
Schematic diagrams of a PGA conduit containing two barrels of DABLs seeded with 3 × 10^6^ BMSCs (conduit group) and a 20 mm long autologous nerve graft (autograft group). In the conduit group, the sural vessel pedicle was attached to the outer surface of the PGA conduit.

We created a nerve conduit using a Lewis rat sciatic nerve model with a 20 mm interstump gap. A 23 mm long capillary permeable tube (inner diameter: 3 mm) made of woven PGA fibers (the outer cylinder portion of Nerbridge^®^) was used. DANBLs were created from sciatic nerves of Dark Agouti rats (which have major histocompatibility mismatch with Lewis rats) using the surfactant extraction method [52,55]. BMSCs taken from bone marrow of femurs and tibias of Lewis rats were cultured over five passages. Two 20 mm long DANBLs were transplanted into each chamber. Three million BMSCs were transplanted into the two DANBLs in the chamber. Each PGA conduit was transplanted between the transected sciatic nerve stumps, leaving a 20 mm gap. Pedicled sural vessels were harvested from the lower hind limb, tuned to the posterior femoral region and attached to the outer surface of the PGA conduit (conduit group). As a control, a 20 mm long sciatic nerve segment was harvested, the proximal–distal direction was reversed, and the segment was transplanted between the transected proximal and distal sciatic nerve stumps (autograft group) (Figure 4). From the electrophysiologic and histomorphometric studies, nerve regeneration through the conduit group was considered to be about 70–90% of that through the autograft group at 24 weeks. However, at 12 weeks, the autograft group outperformed the conduit group significantly in all electrophysiologic and morphohistometric parameters [8]. The rate of nerve regeneration through an autologous nerve graft was significantly faster than that through our conduit [8]. This may be a limitation of our nerve conduits in clinical applications.

## 9. Summary and Discussion

**Figure 5:**
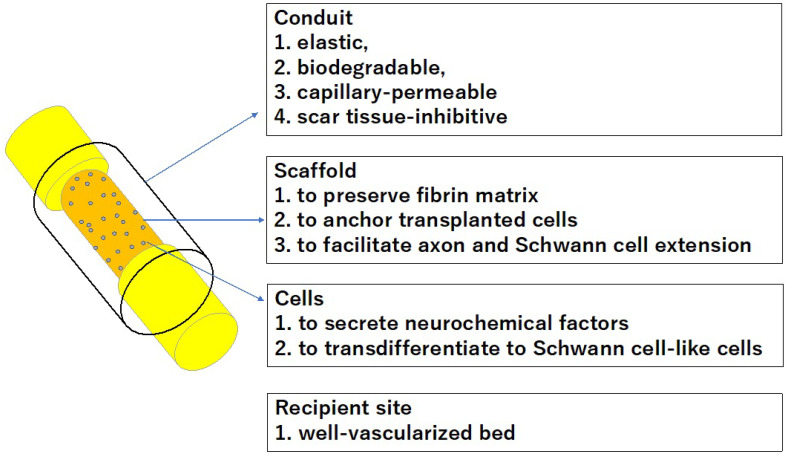
Creation of an artificial nerve conduit with a long interstump gap.

Artificial nerves are used to bridge a short sensory nerve gap. The creation of nerve conduits that can be an alternative to autologous nerve grafts spanning a long interstump gap remains confounded by many problems yet to be solved. Some conduits are used to facilitate or augment nerve sutures [62,63], prevent painful neuroma formation [64,65], or minimize adhesion between nerves and surrounding tissue [66], rather than to increase the number and diameter of regenerated axons or the distance across which axons regenerate. Artificial nerves spanning a long nerve gap can solve the biggest enduring drawback of autologous nerve grafts, which is functional deficit in the recipient sites of nerve grafts. In creating an artificial conduit connecting nerve stumps spanning a long gap, fibrin matrix formation and capillary extension within the long chamber space is crucial. Capillary permeable nerve conduits need some substrate to maintain the fibrin matrix inside the chamber. Capillary permeable tubes must be transplanted in well-vascularized tissue. Porous structures of permeable tubes are associated with the risks of scar tissue invasion into the chamber space and leakage of neurochemical factors and transplanted stem cells from the chamber. Thus, further research is needed to create conduits that minimize leakage of neurochemical factors and transplanted cells from the chamber space and inhibit scar tissue invasion yet maximize capillary extension into the tubular lumen (Figure 5).

DANBLs originate from nerve tissue, which may be structurally favorable for Schwann cell migration and axon extension. Fibrin matrix is formed in the honeycomb structures of DANBLs, which may elongate the distance across which the fibrin matrix is formed in the conduits [8]. In addition, the basal lamellae contain some adhesion molecules such as laminin that may facilitate axon regeneration [6,7,8,60]. The creation of artificial nerve scaffolds which have similar functions to DANBLs remains a great challenge. At the same time, the conduits must be strong enough to avoid collapse but be elastic enough to accommodate motion of the joints.

Transplantation of stem cells in nerve conduits may have facilitated nerve regeneration caused by transdifferentiation of these stem cells into Schwann cell-like cells and/or the secretion of growth factors. Further study is necessary to identify if the transplanted stem cells can transdifferentiate into cells functionally like Schwann cells. We also need to investigate which stem cell class is the best for peripheral nerve regeneration from the perspective of cell differentiation capability and endocrine function (Figure 5).

**Figure 6:**
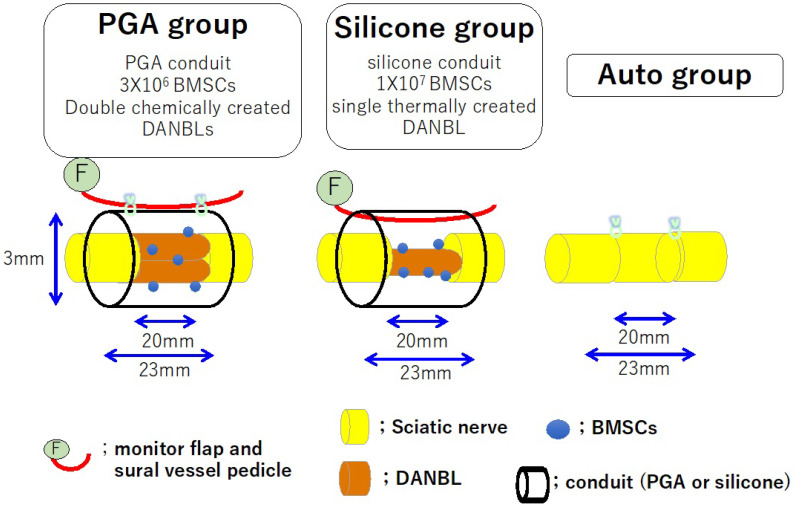
In the PGA group, two pieces of 20 mm long DANBLs (created using chemical surfactants) seeded with 3 × 10^6^ BMSCs was transplanted in a 23 mm long PGA conduit. A sural vessel pedicle was attached to the outer surface of the conduit.

We are interested in the comparison of nerve regeneration in a capillary permeable tube (a PGA tube) with that in a noncapillary permeable tube (silicone tube). Nerve regeneration through a 20 mm long silicone tube containing a sural vessel pedicle, thermally created DANBLs seeded with 1 × 10^7^ BMSCs (silicone group) [6,7], was compared with that through a sural vessel pedicle-attached 20 mm long PGA meshed tube (the outer cylindrical part of Nerbridge^®^) containing chemically created DANBLs seeded with BMSCs (PGA group) (Figure 6) [8]. The nerve regeneration of the silicone group seemed to be slightly better than that of the PGA group, although a direct simple comparison was impossible. The difference might have resulted from the different creation methods used for the DANBLs (thermal vs. chemical) or the different numbers of the transplanted BMSCs. The intrachamber vascular pedicle might have acted as a scaffold for axon extension in the silicone group. Fibrin matrix formation might have been better in the nonpermeable tubes (silicone group) than in the permeable tubes (PGA group) partly because some fibrin matrix and neurochemical factors might have leaked from the chamber space in the PGA group. This might indicate the importance of creating an environment for nerve regeneration that is separate from the surrounding tissue to obtain good nerve regeneration through the conduit. Certainly, the use of capillary permeable tubes makes intrachamber nerve regeneration difficult. However, the nerve regeneration distance would not be elongated without additional vascular supply.

Peripheral nerve regeneration is influenced not only by the local environment at the injured site, but also by the central nervous system, especially the spinal motor neurons or dorsal root ganglions. Because no ribosomes exist in the axons, protein cannot be produced within the axons. Cell organelles and cell structural proteins, which are needed to develop axons at the site of nerve injuries, are produced in the neurons and transferred peripherally through axonal flow by the motor proteins along the microtubulins [67]. The rate of peripheral nerve regeneration may also be influenced by the rate of axonal flow. We must develop methods to facilitate the rate of axonal flow and activate neurons to obtain successful outcomes from artificial nerve conduits.

In the silicone group, a 20 mm long DANBL (created using the thermal method) seeded with 1 × 10^7^ BMSCs was transplanted in a 23 mm long silicone conduit. A sural vessel pedicle was passed through the conduit lumen.

The auto group consisted of a 20 mm long autologous nerve graft model.
